# A Case of Ovarian Clear Cell Carcinoma Simultaneously Producing Parathyroid Hormone-related Protein and Granulocyte Colony-Stimulating Factor

**DOI:** 10.4021/wjon2010.06.214w

**Published:** 2010-05-19

**Authors:** Masayuki Futagami, Yoshihito Yokoyama, Moe Wakui, Ryousuke Taniguchi, Tsuyoshi Higuchi, Hideki Mizunuma

**Affiliations:** a Department of Obstetrics and Gynecology, Hirosaki University Graduate School of Medicine, 5-Zaifu-cho, Hirosaki, Aomori 036-8562, Japan

**Keywords:** Clear cell carcinoma, Hypercalcemia, Increase of leukocytes, Parathyroid hormone-related protein, Granulocyte colony-stimulating factor

## Abstract

We describe the first report of an ovarian clear cell carcinoma simultaneously producing parathyroid hormone-related protein (PTHrP) and granulocyte colony-stimulating factor (G-CSF). A 64-year-old woman complained of general fatigue, loss of appetite, nausea, vomiting and constipation. The results of blood and biochemistry tests were white blood cell count of 21,060 /ml and calcium of 18.0 mg/dl, indicating an increase in the number of leukocytes and hypercalcemia. A computerized tomography scan showed a tumor in the lower abdomen with a maximum diameter of 16 cm and containing both cystic and solid parts. There was a remarkable elevation of the tumor marker CA 19-9, to 1611 IU/ml, and serum level of PTHrP was elevated to 25.9 pmol/ml. The PTH-intact level was 14 pg/ml, which was at the lower limit of the normal range. In addition, the G-CSF level was also elevated to 73 pg/ml (normal range: <38 pg/ml). Since hypercalcemia caused by tumor PTHrP production was suspected, and as this required elimination of the primary disease, extirpation of the tumor was carried out. Serum calcium levels promptly returned to 11.1 mg/ml on the first day following surgery, and PTHrP also dropped to its normal level on the same day. Histological and immunohistochemical examinations revealed that the tumor was clear cell adenocarcinoma which was partially positive for PTHrP and positive for G-CSF, indicating the tumor simultaneously producing PTHrP and G-CSF.

## Introduction

Hypercalcemia is observed in 10-15% of malignant tumor cases. Malignancy-associated hypercalcemia can be basically divided into two subtypes; local osteolytic hypercalcemia (LOH) caused by local bone erosion and humoral hypercalcemia of malignancy (HHM) by systemic bone loss induced by other causes. The most common cause of HHM is parathyroid hormone-related protein (PTHrP) produced by tumor cells [1]. Among malignant ovary neoplasms, clear cell carcinoma is one that is most frequently associated with hypercalcemia [2], whereas ovarian cancer complicated by hypercalcemia generally tends to have a poor prognosis [3]. Furthermore, leukocytosis is associated with some malignant neoplasms, in which granulocyte colony-stimulating factor (G-CSF) is produced by tumor tissue.

We describe a case of ovarian clear cell carcinoma with hypercalcemia and leukocytosis, presumably resulting from simultaneous secretion of PTHrP and G-CSF from an ovarian clear cell carcinoma. We present this case with a literature-based discussion.

## Case Report

A 64-year-old woman, gravida 3, para 2, without significant medical history visited a local physician in January 2010 with chief complaints of general fatigue, loss of appetite, nausea, vomiting and constipation. An abdominal computerized tomography (CT) scan was carried out to identify the cause, and this detected a pelvic tumor, following which the patient was referred to our clinic due to suspected gynecological disorder. The results of blood and biochemistry tests at her first visit were white blood cell count (WBC) of 21,060 /ml, C-reactive protein (CRP) of 13.3 mg/dl, blood urea nitrogen (BUN) of 40 mg/dl, creatinine (Cre) of 1.98 mg/dl, and calcium of 18.0 mg/dl, indicating an increase in the number of leukocytes, elevated CRP, impaired renal function, and hypercalcemia. Due to apparent hypercalcemia and renal failure the patient was hospitalized and given medical treatment including fluid replacement and administration of diuretics and corticosteroid.

A CT showed a tumor in the lower abdomen with a maximum diameter of 16 cm and containing both cystic and solid parts. Distant metastasis, and pelvic and paraaortic lymph node enlargement were not clearly observed. There was a remarkable elevation of the tumor marker CA 19-9, to 1,611 IU/ml, and serum level of PTHrP was elevated to 25.9 pmol/ml. The PTH-intact level was 14 pg/ml, which was at the lower limit of the normal range. In addition, the G-CSF level was also elevated to 73 pg/ml (normal range: <38 pg/ml). Severe fatigue, loss of appetite, and constipation were observed, whereas there was no disturbance of consciousness or any electrocardiogram abnormality found. Since hypercalcemia caused by tumor PTHrP production was suspected, and as this required elimination of the primary disease, total abdominal hysterectomy, bilateral sapingo-oophorectomy, and omentectomy were carried out on the second day of hospitalization. Serum calcium levels promptly returned to 11.1 mg/ml on the first day following surgery, and PTHrP also dropped to its normal level on the same day. Serum BUN and Cre dropped to 23 mg/dl, 1.58 mg/dl, respectively, on day 3 post-surgery. PTH-intact, which was at a low level pre-surgery, rapidly increased to 135 pg/ml on day 15 post-surgery. This was likely due to hungry bone syndrome, which is an elevation in response to the rapid drop of the PTHrP level after surgery [4], and is treated by replacement therapy with oral administration of vitamin D. Finally, the patient’s serum calcium level was 9.1 mg/dl on day 15 post-surgery.

Based on the macroscopic observations of the tumor, it was derived from the right ovary, had a smooth surface, and was neoplasm which weighed 2 kg. The tumor contained brown liquid inside and had a soft and opal-colored cross-section in the solid part.

**Figure 1:**
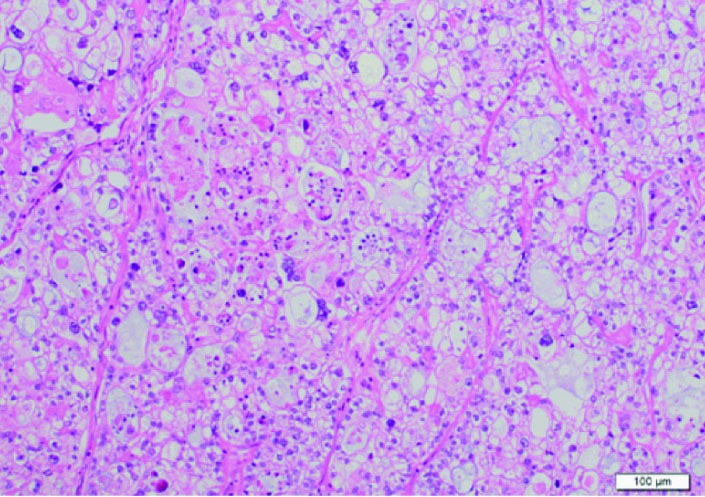
Histological examination. Atypical cells with clear cytoplasm and anisonucleosis were growing hyperplastically in sheet-like and cystic structures. Its finding is identical to clear cell carcinoma.

**Figure 2:**
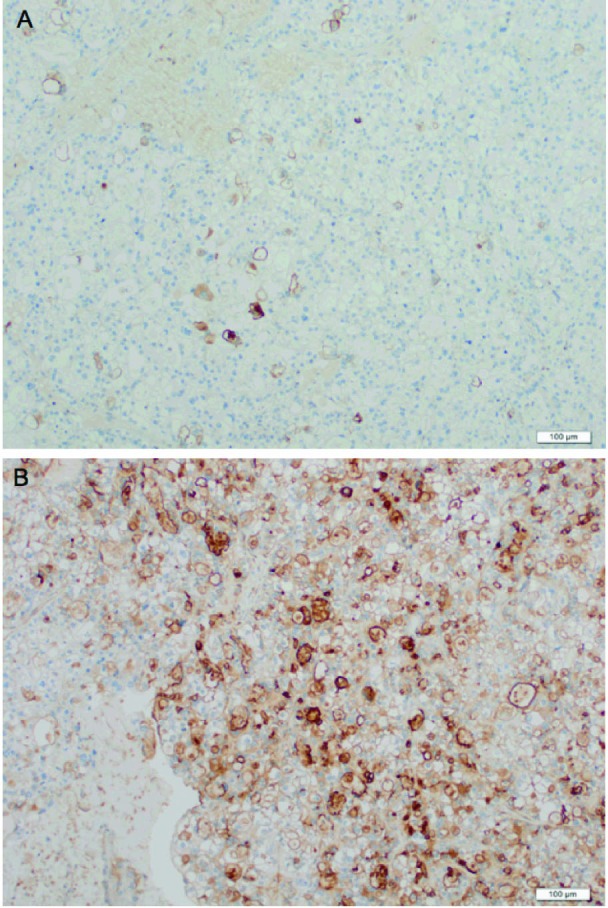
Immunohistochemical staining of the tumor. Atypical cells were partially positive for PTHrP (A) and positive for G-CSF (B).

According to histopathological examinations (Fig. 1), atypical cells with clear cytoplasm and anisonucleosis were growing hyperplastically in sheet-like and cystic structures. The patient was diagnosed with clear cell adenocarcinoma. Atypical cells were partially positive for PTHrP (Fig. 2A) and positive for G-CSF (Fig. 2B). The case was considered to be clear cell carcinoma producing PTHrP and G-CSF. The patient had negative peritoneal cytology and was staged pT1a.

## Discussion

Hypercalcemia has been reported to occur in 10-15% of malignant tumor cases, and is the most common malignancy-associated endocrinological disorder. Tumors causing hypercalcemia are most likely squamous cell carcinomas of the lung, which, together with breast cancer and myeloma, cause more than 50% of cases and are followed by other squamous cell carcinomas and renal cell carcinoma [5].

Malignancy-associated hypercalcemia can be basically divided into two subtypes: LOH caused by local bone erosion and HHM by systemic bone loss induced by other causes. The most common cause of HHM is PTHrP produced by tumor cells [1]. Hypercalcemia symptoms include those related to the gastrointestinal tract, such as nausea, vomiting, and constipation; neuropsychiatric symptoms such as depression; and various other symptoms such as dehydration and renal failure. The present case expressed symptoms of general fatigue, loss of appetite, nausea, vomiting and constipation. The previous physician initially suspected an infectious disease because of the high white blood cell count, suggesting the importance of recognizing the association between a tumor and hypercalcemia as well as leukocytosis.

Hypercalcemia is known to be associated with about 5% of gynecological malignant diseases, and cases involving various organs such as the uterus, ovaries, vulva, and vagina have been reported [6]. It is most commonly associated with ovarian cancer, and the majority of cases are clear cell carcinomas and small cell carcinomas. Savvari et al. [7] reported in a review that 11 out of 22 cases (50%) of hypercalcemia associated with ovarian tumor were clear cell carcinomas, and the present case was also a clear cell carcinoma.

In most cases of malignancy-associated hypercalcemia, the serum calcium level temporally drops in response to medical treatment, which includes fluid replacement and administration of diuretics, calcitonin preparations, bisphosphonate preparations, and corticosteroids. Although bisphosphonate preparations are extremely effective especially for malignancy-associated hypercalcemia, their action is relatively slow and takes several days to reach its peak effect [8]. Therefore, hemodialysis with low calcium dialysate is an option for highly urgent cases. In any case, it is critical to treat the primary disease by surgery or chemotherapy [8]. The present case also exhibited hypercalcemia and an elevated level of serum PTHrP, which only partially responded to medical treatment, and rapidly returned to a normal level after extirpation of the ovarian tumor. Furthermore, immunohistochemical investigation of the surgical specimen revealed that the tumor tissue was partially positive for PTHrP, suggesting that hypercalcemia was caused by PTHrP produced by the ovarian tumor. Before extirpation of the tumor, PTHrP caused accelerated bone loss, enhanced renal calcium reabsorption, and reduced renal concentrating ability, resulting in elevated levels of serum calcium, BUN and Cre. PTH secretion was previously suppressed and elevated after extirpation of the tumor, presumably in response to PTHrP activity, which was previously high and disappeared after extirpation of the tumor. It was not clear why the serum calcium level remained within the normal range even though PTH secretion was accelerated. However, the serum PTH level gradually fell during the follow-up monitoring.

Although ovarian cancers with hypercalcemia have been reported to have poorer prognoses than those without [9], there is no clear association between serum calcium levels and prognosis. In addition, there appears to be some association between the serum PTHrP level and prognosis, but one report did not find a statistically significant correlation [7]. However, due to the limited number of cases it is not clear whether examination upon prognosis is adequate. Furthermore, the histologic types of ovarian cancer expressing hypercalcemia are mostly clear cell carcinoma and small cell carcinoma with poor prognoses. Consequently, ovarian cancer associated with hypercalcemia should be monitored very carefully during post-surgical follow-up. Furthermore, particular attention needs to be paid in cases of malignancy-associated hypercalcemia, as its sudden onset may cause renal failure and exacerbate systemic conditions.

Some malignant tumors in various organs such as the lungs, bladder, pancreas, stomach and colon, are known to produce G-CSF [10-14], whereas reports on G-CSF production are very rare in the field of gynecology. In the present case G-CSF production from the tumor cells was suspected because leukocytosis (neutrocytosis) was observed before surgery. Consequently, the serum G-CSF level was measured and found to be high. Furthermore, the leukocyte count returned to normal shortly after extirpation of the tumor and a surgical specimen of the tumor had positive immunohistochemical staining for G-CSF, suggesting that the tumor was a G-CSF producing one. G-CSF producing tumors are characterized by 1) leukocytosis, 2) elevated G-CSF, 3) rapid return to a normal leukocyte count following extirpation of the tumor, and 4) evidence of G-CSF production in the tumor [15], all of which were found in the present case. G-CSF producing tumors generally tend to have poor prognoses [13] and proliferation of tumor cells due to G-CSF has been reported in some experiments using cells from malignant ovarian tumors and other malignant tumors [16-18]. However, other reports have concluded that its effect was almost negligible at concentrations clinically used for treatment of diseases such as neutropenia [19, 20].

To our knowledge, this is the first report of a gynecologic tumor simultaneously producing PTHrP and G-CSF. Although the interaction between these two produced substances is unknown, the case should be followed up carefully because of the involvement of two factors with a generally poor prognosis and clear cell carcinoma.

In conclusion, we identified a case of ovarian clear cell carcinoma associated with hypercalcemia and leukocytosis. To our knowledge, no case with both of these has been reported to date, and this case was at least the first to be confirmed by a blood test and immunohistochemical examination. Production from a tumor should be suspected in cases with gastrointestinal symptoms such as nausea, vomiting and constipation, obvious dehydration, leukocytosis with no identifiable source of infection, together with a tumor in the pelvis. Particular caution should be taken with PTHrP and G-CSF producing tumors and they must be treated very carefully, as they have been reported to have poor prognoses.
